# Quality of life and its predictors among adult patients with haemophilic arthropathy. An observational study

**DOI:** 10.1186/s12891-021-04319-0

**Published:** 2021-05-15

**Authors:** Roberto Ucero-Lozano, José Antonio López-Pina, Alba Ortiz-Pérez, Rubén Cuesta-Barriuso

**Affiliations:** 1 Department of Physiotherapy, European University of Madrid, Madrid, Spain; 2 Department of Basic Phycology and Methodology, University of Murcia, Murcia, Spain; 3 Health Psychologist, Free Exercise of the Profession, Madrid, Spain; 4 Department of Physiotherapy, University of Murcia, Murcia, Spain; 5 Royal Victoria Eugenia Foundation, Madrid, Spain

**Keywords:** Haemophilia, Quality of life, Joint pain, Joint damage, Kinesiophobia, Catastrophism

## Abstract

**Background:**

Recurrent hemarthrosis that begin in childhood lead to progressive joint deterioration. Patients with haemophilia have chronic pain, functional disability and a reduced perception of health-related quality of life.

**Purpose:**

To analyse the perceived quality of life of adult patients with haemophilic arthropathy and its relationship with pain, joint condition, kinesiophobia and catastrophism.

**Methods:**

Eighty-three adult patients with haemophilia were included in this multicentre, cross-sectional, descriptive study. Perceived quality of life (*36-Item Short Form Health Survey*), perceived usual and maximum pain (visual analogue scale), joint condition (*Haemophilia Joint Health Score*), kinesiophobia (*Tampa Scale of Kinesiophobia*) and catastrophism (*Pain Catastrophizing Scale*) were assessed. Sociodemographic, clinical and therapeutic variables and drug consumption for pain control were collected. Descriptive statistics used means and standard deviations. The correlation of quality of life with the dependent variables was calculated with the Pearson correlation test. The differences in quality of life as a function of the binomial variables were calculated with Student’s t-test for independent samples.

**Results:**

Physical component of quality of life perceived by patients with hemophilia is lower than Spanish population (30.51 VS 48.85). Regarding the mental component, patients with hemophilia showed higher values (56.07 VS 49.97). Catastrophism correlated (*p* < .05) with all items of quality of life questionnaire. Kinesiophobia correlated (*p* < .05) with all items of quality of life except to role-emotional (*r* = -.18; *p* > .05). Habitual and maximal joint pain correlated with all items except to role-emotional (*r* = − .19 and *r* = − .09, respectively) and mental component score (*r* = − .16 and *r* = − .07, respectively). Catastrophism and weekly drug intake were inversely correlated with quality of life. Age was positively correlated with perceived quality of life. There were differences in quality of life as a function of the severity of haemophilia and the intake of drugs for pain control.

**Conclusions:**

The perceived quality of life of adult patients with haemophilia is worse than that of the Spanish population. Pain, kinesiophobia, catastrophism, haemophilia severity and the intake of pain-control medication influence the quality of life of these patients.

## Plain English summary

This study identifies the clinical and therapeutic factors that affect the quality of life of adult patients with degenerative haemophilic arthropathy.The perceived quality of life of adult patients with joint damage and the factors that influence the quality of life of adult patients with haemophilic arthropathy were observed.A total of 83 patients with a rare disease and an advanced degree of joint damage were recruited from across the country. Different psychosocial, clinical and therapeutic variables common to all patients with haemophilia were assessed.Pain, kinesiophobia, catastrophism and weekly drug intake are the variables that most influence quality of life. A quality-of-life-based approach should be a fundamental aspect in the treatment of these patients.

## Background

The severity of haemophilia depends on the clotting factor in functional plasma and is categorized as mild (5–40 % FVIII), moderate (1–5 %) and severe (< 1 %) [1]. In severe haemophilia, bleeding in the musculoskeletal system with mild or spontaneous trauma is common. Haemophilia A occurs worldwide with an estimated incidence of 1 in 5,000 male births [2]. Prophylactic treatment has been shown to be effective for reducing bleeding and its consequences [3], but because of its cost and associated discomfort, not all patients choose it [4]. The development of antibodies to treatment (inhibitors) is the main clinical complication experienced by these patients [5].

The characteristic clinical manifestation of haemophilia is the development of musculoskeletal haemorrhages, mainly joint bleeding. In 70–80 % of cases, hemarthrosis is located in the knees, elbows and ankles [6]. The lower limbs have a higher incidence due to the pressure and load transmission components that they support [7]. Recurrent joint haemorrhagic episodes that begin in childhood lead to progressive joint deterioration [8], which causes changes in the cartilage, bone, synovium and vessels. In the final phase of joint damage, a degenerative and chronic lesion known as haemophilic arthropathy is established.

Arthropathy causes changes in joint condition; decreased range of motion; proprioceptive, biomechanical and gait disturbances; and atrophy of the periarticular musculature [9]. As a consequence, patients report chronic pain and loss of functionality [10], which leads to a disability that negatively influences the perceived quality of life (QoL) of these patients [9].

Quality of life includes individuals’ perception regarding their position in life within the context and values of the culture in which they live and in relation to their goals, expectations, standards and concerns [11]. Patients with haemophilia have chronic pain, psychological problems, functional disability and a reduced perception of health-related quality of life [12]. Variables such as joint deterioration of haemophilic arthropathy [13], haemophilia severity [14] and joint pain [15] are related to a worse perceived quality of life in these patients. Chronic pain, such as that experienced by patients with haemophilic arthropathy, increases pain-related fear and avoidance behaviours in response to aversive stimuli. Avoidant behaviours usually involve activity restrictions and interference with activities of daily living [16]. One of these fears is kinesiophobia, defined as an excessive, irrational and debilitating fear of physical movement and activity due to the assumption of increased pain or major injury [17]. A high level of kinesiophobia is associated with a worse health-related quality of life, regardless of the location of the lesion [18].

Prophylaxis versus on demand treatment in adults with severe hemophilia and arthropathy decrease bleeding, pain, and better joint health, activity, satisfaction, and perception of quality of life [19] Based on the above evidence stressing on the importance of prophylaxis treatment for patients with hemophilic arthropathy, a hypothesis was proposed whereby the patients with hemophilia had a similar perception of quality of life with respect to the Spanish population without hemophilia. Another hypothesis of this study is that pain, kinesiophobia and catastrophism negatively influence the quality of life of these patients.

The main objective of this study was to assess the current perceived quality of life in a population of adult patients with haemophilia in different regions of Spain. The secondary objectives were (i) to analyse the correlation of perceived quality of life with the patients’ perceived pain, joint condition, kinesiophobia, catastrophism and coping strategies; (ii) to assess the relationship of quality of life with age and clinical variables (severity, type of treatment and development of inhibitors); and (iii) to identify possible modifiable aspects to improve the quality of life of these patients.

## Methods

### Setting

This was a multicentre, cross-sectional, descriptive study. Data were collected at the national level from March 2018 to October 2019, with the purpose of collecting information on the health status and perceived quality of life of patients with haemophilic arthropathy. The data were collected through face-to-face personal interviews and physical assessments.

### Patients

Patients were recruited from nine patient associations throughout Spain (Bilbao, Madrid, Malaga, Murcia, Santiago de Compostela, Valencia, Valladolid, Vigo, Zaragoza). Patients who were interested in participating in the study and who met the selection criteria contacted the researchers through their respective associations.

The inclusion criteria for the study were as follows: patients with haemophilia A or B; patients with a diagnosis of haemophilic arthropathy (more than 3 points on the *Haemophilia Joint Health Score (HJHS)*) [20]; and patients over 18 years of age. The exclusion criteria were as follows: patients who did not complete all the measurement instruments or who were unable to understand them; patients who had hemarthrosis in the four weeks prior to the study; patients who were unable to ambulate without the help of a third person; and patients who did not sign the informed consent document.

### Assessments

A record of relevant clinical and sociodemographic data was prepared, and the following questionnaires were used:

- Perceived quality of life was measured with the *36-Item Short Form Health Survey* (SF-36) scale [21]. This self-administered scale consists of 36 items in eight domains: physical functioning, limitations due to physical problems, physical pain, social role or function, mental health, limitations due to emotional problems, vitality and general perception of health.
- Joint condition was measured with the HJHS [20]. This scale, designed specifically for patients with haemophilia, assesses the joint condition of patients with haemophilic arthropathy in the knees, ankles and elbows. This tool assesses eight items: inflammation, duration of inflammation, pain-related muscle atrophy, strength, crackles and loss of flexion and extension movements. The scoring range is from 0 (no joint damage) to 20 points (maximum joint damage) per joint. The maximum joint score is 120 points, to which the gait score is added (0–4 points range).
- Perceived pain was measured with a visual analogue scale. The patients were asked about their maximum pain and usual pain in the week prior to the study. This scale has a range of 0 to 10 points (from no pain to maximum perceived pain).
- Catastrophism was assessed with the *Pain Catastrophizing Scale *[22]. This scale is composed of 13 items rated on a scale from 0 to 4 points (a lower score indicates lower catastrophism).
- The *Tampa Scale of Kinesiophobia* (TSK-11) was used to assess patients’ fear of moving or engaging in physical activity [23]. We used the Spanish version [24] of this self-administered instrument, which consists of 11 items rated on a 4-point Likert scale. The total score ranges from 11 to 44, with higher values corresponding to a greater fear of being injured again by the movement. Scores of more than 27 points are considered high [25]. The minimum detectable change of this instrument is 5.6 [26].

### Analysis

Data analysis was performed with the statistical package SPSS Statistics version 25 (IBM, Inc., Armonk, NY). Descriptive statistics were calculated using central tendency (mean) and dispersion (standard deviation). The correlation between perceived quality of life and the dependent variables was determined using the Pearson correlation test. Using a one-way ANOVA test, differences in perceived quality of life were analysed as a function of haemophilia severity, treatment type and the development of inhibitors. All results were considered significant at a level < 0.05.

### Local approvals

The study was approved by the Ethics Committee of the Virgen de la Arrixaca University Hospital (id. 2020-2-9-HCUVA). The patients signed written informed consent documents before beginning the study assessments. The study was registered with the International Registry of Clinical Trials www.clinicaltrials.gov (id. NCT03499522).

## Results

Eighty-three patients were recruited for this study. The mean age was 43.87 years (DT: 10.10), with a mean height of 1.79 m (DT: 0.07), with a mean weight of 85.93 kg (DT: 13.09) and a mean body mass index of 26.62 kg/m2 (DT: 3.48). The majority of the patients had a diagnosis of haemophilia A (83.1 %), received prophylactic treatment (28.9 %) and had a severe phenotype of the disease (85.5 %). 51 patients (61.44) were coinfected with both HIV and HCV. Regarding the consumption of drugs for pain control, the majority of the subjects (69.9 %) used pain medications, with a mean frequency of 4.13 (SD: 2.99) days per week. The most commonly used drugs were celecoxib (32.8 %), NSAIDs (27.6 %), etoricoxib (17.2 %), etoricoxib and celecoxib (8.6 %), celecoxib and NSAIDs (8.6 %) and paracetamol (5.2 %). (Table 1).

**Table 1:** Sample demographics and clinical characteristics, presented as mean (standard deviation)

Variables	Items	Mean (SD)
Age	Age (years)	43.87 (10.10)
Intake pain medication	Days/week	4.13 (2.99)
Joint health	Ankle joint health (range 0–40)	20.53 (5.35)
	Knee joint health (range 0–40)	17.83 (7.17)
	Elbow joint health (range 0–40)	15.65 (5.50)
	Joint health (range 0-124)	55.83 (16.06)
Catastrophism	Perceived catastrophism	10.12 (8.65)
Kinesiophobia	Perceived kinesiophobia	30.60 (7.24)
Quality of life	Physical functioning (range 10–30)	33.37 (22.11)
	Role-physical (range 4–8)	73.13 (24.12)
	Body pain (range 2–12)	46.12 (21.43)
	General health (range 5–25)	30.12 (17.96)
	Vitality (range 4–24)	66.81 (9.89)
	Social functioning (range 2–10)	67.05 (25.01)
	Role-emotional (range 3–6)	95.01 (13.93)
	Mental health (range 5–30)	72.92 (10.36)
	Physical component score (range 0–40)	30.51 (8.62)
	Mental component score (range 0–40)	56.07 (5.71)
Joint pain	Habitual joint pain (range 0–10)	4.48 (1.64)
	Maximum joint pain (range 0–10)	7.95 (1.37)
		n (%)
Clinical variables	Type of haemophilia (A/B)	69 / 14 (83.1 / 16.9)
	Severity of haemophilia (moderate/severe)	12 / 71 (14.5 / 85.5)
	Type of treatment (on demand/prophylactic)	24 / 59 (28.9 / 71.1)
	Development of inhibitors (no/yes)	70 / 13 (84.3 / 15.7)
	Drug use (no/yes)	25 / 58 (30.1 / 69.9)

Catastrophism was negatively correlated with all domains of perceived quality of life (*p* < .05). The use of drugs for pain control was negatively correlated with all domains (*p* < .001) except for the total mental health score (*r* = -.14; *p* = .19). Kinesiophobia was negatively correlated with all domains of quality of life (*p* < .05) except for emotional role (*r* = -.18; *p* = .09).

The maximum perceived pain was negatively correlated (*p* < .05) with most of the domains of quality of life except emotional role (*r* = -.09; *p* = .38) and total perceived mental health (*r* = -.07; *p* = .48). The usual pain perceived by the patients was negatively correlated (*p* < .05) with all domains except the emotional role and the total perceived mental health.

The overall joint condition was negatively correlated only with mental health (*r* = -.25; *p* = .01) and the total mental health score (*r* = -.22; *p* = .04). Elbow joint deterioration presented an inverse correlation with social functioning (*r* = -.22; *p* = .04) and mental health (*r* = -.27; *p* = .01). The degree of ankle arthropathy was negatively correlated with the total perceived mental health (*r* = -.22; *p* = .04).

Finally, age was the only variable that was positively correlated with the domains of body pain (*r* = .43; *p* < .001), general health (*r* = .37; *p* < .001), vitality (*r* = .32; *p* < .01), social functioning (*r* = .37; *p* < .01) and total perceived physical health (*r* = .30; *p* < .01). (Table 2).

**Table 2:** Correlations between quality of life and joint pain, joint health, catastrophism, kinesiophobia, age and weekly drug intake

Quality of life items	Habitual joint pain	Maximum joint pain	Knee health	Ankle health	Elbow health	All joint health	Catastrophism	Kinesiophobia	Age	Weekly drug intake
Physical functioning	− 0.383**	− 0.444**	− 0.022	0.033	− 0.194	− 0.067	− 0.350**	− 0.333**	0.001	− 0.506**
Role-physical	− 0.721**	− 0.575**	− 0.123	− 0.077	− 0.123	− 0.129	− 0.748**	− 0.628**	0.174	− 0.570**
Body pain	− 0.635**	− 0.681**	− 0.103	− 0.131	− 0.185	− 0.156	− 0.658**	− 0.644**	0.436**	− 0.528**
General health	− 0.494**	− 0.519**	− 0.017	− 0.025	− 0.170	− 0.071	− 0.491**	− 0.515**	0.375**	− 0.437**
Vitality	− 0.374**	− 0.398**	− 0.113	− 0.107	− 0.211	− 0.165	− 0.417**	− 0.454**	0.329**	− 0.447**
Social functioning	− 0.559**	− 0.477**	− 0.069	− 0.116	− 0.220*	− 0.149	− 0.528**	− 0.503**	0.371**	− 0.382**
Role-emotional	− 0.197	− 0.096	− 0.187	− 0.189	− 0.030	− 0.174	− 0.218*	− 0.185	− 0.238*	− 0.236*
Mental health	− 0.313**	− 0.316**	− 0.192	− 0.198	− 0.276*	− 0.259*	− 0.498**	− 0.420**	0.195	− 0.391**
Physical component score	− 0.657**	− 0.671**	− 0.033	− 0.008	− 0.191	− 0.082	− 0.626**	− 0.602**	0.306**	− 0.597**
Mental component score	− 0.160	− 0.077	− 0.176	− 0.226*	− 0.158	− 0.223*	− 0.252*	− 0.223*	0.103	− 0.144

*Significant difference (*p* < .05)

** Significant difference (*p* < .001)

The patients who did not take pain-control drugs showed better perceived quality of life in all domains (*p* < .01) except total perceived mental health (*p* = .09; 95 % CI: -0.41–4.96). Patients with severe haemophilia indicated worse perceived quality of life (*p* < .05) in all domains assessed except in total perceived mental health (*p* = .50; 95 % CI: -2.36–4.76). Patients without inhibitors showed better physical functioning (*p* < .01; 95 % CI: 4.40–30.04), physical role (*p* = .01; 95 % CI: 4.10–32.14) and total perceived physical health (*p* = .01; 95 % CI: 1.17–11.24) than those who had not developed antibodies (Table 3).

**Table 3:** Relationship between quality of life and inhibitor development, treatment type, haemophilia severity and the consumption of drugs for pain control

Quality of life items	Inhibitors			Treatment type			Haemophilia severity			Drug intake		
	F	MD	95 %CI	F	MD	95 %CI	F	MD	95 %CI	F	MD	95 %CI
Physical functioning	0.15ª	17.22 *	-32.56 - -1.88	3.29	-6.68	-17.30–3.93	0.54	25.28 **	12.64–37.91	0.11	21.50 **	12.03–30.96
Role-physical	1.18ª	18.12 *	-35.41 - -0.84	2.66ª	-13.06	-24.39 - -1.73	3.15	20.40 **	6.01–34.78	13.63ª	27.80 **	20.32–35.37
Body pain	0.00	8.62	-21.44–4.19	0.13	-8.38	-18.61–1.83	0.003	17.78 **	4.98–30.58	0.26	27.36 **	19.06–35.65
General health	2.75	2.33	-13.18–8.51	1.27	-4.28	-12.94–4.36	2.32	16.81 **	6.21–27.40	0.72	15.85 **	7.99–23.71
Vitality	0.70	2.59	-8.55–3.35	5.01	0.49	-4.30–5.28	1.39	7.62 *	1.68–13.57	4.17ª	10.00 **	6.63–13.38
Social functioning	0.47	8.63	-23.63–6.37	0.13	-1.22	-5.66–6.64	0.35	16.79 *	1.61–31.98	0.76	20.88 **	9.82–31.93
Role-emotional	0.35	-0.71	-7.71–9.14	0.17	-1.15	-13.34–10.90	6.17ª	5.83 **	2.30–9.36	14.56ª	7.13 **	2.86–11.40
Mental health	0.09	1.08	-7.34–5.17	0.86	-0.82	-7.90–5.59	6.80ª	7.50 **	3.84–11.16	16.08ª	9.45 **	6.16–12.74
Physical component score	0.31ª	6.20 *	-12.70–0.28	0.51	-3.83	-5.84–4.19	0.11	9.72 **	4.78–14.66	4.39ª	10.60 **	7.63–13.58
Mental component score	0.21	-1.40	-2.03–4.84	1.05	1.23	-7.92–0.25	2.40	1.20	-2.36–4.76	1.44	2.27	-0.41–4.96

ª Mann-Whitney U test; MD: mean difference; 95 % CI: 95 % confidence interval

*Significant difference (*p* < .05)

** Significant difference (*p* < .01)

When analysing which variable influences a greater proportion of the different domains of perceived quality of life (*r* > .5), we observed that the severity of haemophilia influences the patient’s mental health (t (37.28) = 4.14; *r* = .56). The intake of drugs for pain influences physical role (t (80.65) = 7.39; *r* = .63), body pain (t (81) = 6.56; *r* = .58), vitality (t (75.94) = 5.90; *r* = .56) and total perceived physical health (t (64.08) = 7.13; *r* = .66). The type of treatment and the development of inhibitors minimally influenced perceived quality of life (*r* < .30). (Table 4).

**Table 4:** Effect of inhibitor development, treatment type, haemophilia severity and drugs use on the variance

Quality of life items	Inhibitors			Treatment type			Haemophilia severity			Drug intake		
	t	df	r	t	df	r	t	df	r	t	df	r
Physical functioning	2.67	81	0.28	-1.25	81	0.13	3.98	81	0.40 ^b^	4.52	81	0.44 ^b^
Role-physical	2.57	81	0.27	-2.29	81	0.24	2.82	81	0.29	7.39	80.65	0.63 ª
Body pain	1.33	81	0.14	-1.63	81	0.17	2.76	81	0.29	6.56	81	0.58 ª
General health	0.42	81	0.04	− 0.98	81	0.10	3.15	81	0.33	4.01	81	0.40 ^b^
Vitality	0.86	81	0.09	0.16	29.23	0.03	2.55	81	0.27	5.90	75.94	0.56 ª
Social functioning	1.14	81	0.12	− 0.20	81	0.02	2.20	81	0.23	3.75	81	0.38 ^b^
Role-emotional	− 0.16	81	0.01	− 0.34	81	0.03	3.29	70.0	0.36 ^b^	3.34	57.0	0.40 ^b^
Mental health	0.34	81	0.03	− 0.32	81	0.03	4.14	37.28	0.56 ª	5.71	79.81	0.29
Physical component score	2.45	81	0.26	-1.86	81	0.20	3.91	81	0.39 ^b^	7.13	64.08	0.66 ª
Mental component score	− 0.81	81	0.08	0.88	81	0.09	0.67	81	0.07	1.67	81	0.28

ª *r* > .50: the effect explains 25 % of the total variance

^b^*r* > 0.30; the effect explains 9 % of the total variance

## Discussion

The objective of this study was to assess the perceived quality of life of adult patients with haemophilia and to determine the variables that are most highly correlated with quality of life in this patient population. Kinesiophobia, catastrophism, pain perception and drug intake are the variables that are most correlated with perceived quality of life. Patients with moderate haemophilia and those who did not take pain-control drugs had a better perceived quality of life.

Taking as reference the normative values for the healthy Spanish population [27], the physical component of quality of life perceived by adult patients with haemophilia is 18.34 points lower (48.85 VS 30.51). This decrease may be due to the physical limitations caused by haemophilic arthropathy and has been observed in patients with other chronic pathologies [28, 29]. Carroll et al. [30] measured the perceived quality of life of patients with haemophilia in France and England and observed values for the physical component that were up to 10 points higher than those found in our study. This difference between adult patients with haemophilia in Spain and other European countries could be due to the worse joint condition of the patients secondary to the later implementation of prophylactic treatments in Spain. In 2006, only 38 % of patients with moderate and severe haemophilia A received prophylactic treatment in Spain, and values near those in other European countries were not reached until 2013 [31].

Regarding the mental component of perceived quality of life, the patients with haemophilia included in our study showed higher values than the average reported for the Spanish population (56.07 VS 49.97). This difference, despite the development of joint sequelae characteristic of adult patients with haemophilia, may be due to patients’ ability to adapt over the years. The stabilization process, the absence of acute processes with the onset of advanced degenerative damage and compensatory measures to overcome physical limitations could lead to better mental perceived mental health among patients with haemophilia.

Forsyth et al. [32] indicated that 89 % of patients with haemophilia reported experiencing pain that interferes with their daily activities. There is little evidence indicating which drugs to administer for the treatment of pain in patients with haemophilia. However, there seems to be a consensus regarding the use of paracetamol for the control of acute and chronic pain in children and adults with haemophilia [33]. Other drugs commonly administered to patients with haemophilia are strong opioids, cyclooxygenase 2 (COX-2) inhibitors and nonselective NSAIDs. However, in our study, we observed that the most commonly used drug for pain control was celecoxib, followed by NSAIDs. However, the intake of medications is related to a worse perceived quality of life in all areas except in the mental component. In addition, the use of large amounts of drugs is correlated with a worse perceived quality of life.

The patients included in our study had high values for kinesiophobia. This finding coincides with observations for patients with haemophilia that have been reported in previous studies [34, 35]. High levels of kinesiophobia are frequent in subjects with chronic pain and are associated with the maintenance of pain [36]. In addition, we observed negative correlations between kinesiophobia and all items of perceived quality of life except the emotional role. A recent systematic review [37] found a negative correlation between kinesiophobia and perceived quality of life on the items most strongly related to the physical component in patients with musculoskeletal pain. The development of musculoskeletal bleeding causes intense pain in patients with haemophilia that can justify the fear of injury and thereby limits their quality of life.

Holstein et al. [38] reported that 35 % of adults and 8 % of children with haemophilia suffer from chronic pain. The symptoms reported by these patients are similar to those described by other patients with chronic musculoskeletal pain [35]. In our study, we found negative correlations between the intensity of perceived pain, both usual and maximum, and all items of perceived quality of life except for the emotional role and the mental component. Our results are consistent with those reported by Forsyth et al. [32], who indicate that greater pain intensity is related to a worse perceived quality of life. This relationship could be due to the influence of pain on activity and therefore on patients’ perceptions of the more physical components of the assessments.

The inverse relationship between joint condition and quality of life has been described by Davari et al. [39]. The condition of the joints with haemophilic arthropathy was negatively correlated with mental health and the mental components of quality of life. However, we found no correlation between the physical components of quality of life and joint condition. This may be due to the adaptations and compensations that adult patients with haemophilia have made for years to prevent haemophilic arthropathy from limiting their activities of daily living. Over time, those compensations can allow a patient’s joint situation to not influence his or her perceived quality of life.

Forsyth et al. [32] indicated that age was related to a worse perceived quality of life. However, in our study, we found that the older the patients were, the better the quality of life they reported, except for emotional role. This difference in the results could be due to the level of acceptance of the pathology in adult subjects. Experience with the disease, the memory of acute episodes and adaptation to the sequelae and limitations of haemophilic arthropathy may justify the better perceived quality of life of adult patients compared to younger patients. The recurrence of haemarthrosis, the acute processes of synovitis and the development of haemophilia arthropathy, which occur mainly during adolescence and youth, may limit the capabilities and quality of life of younger patients. Acceptance of the pathology has been shown to have an influence on perceived quality of life in patients with chronic pathology [40].

This study aims to increase scientific knowledge regarding the psychosocial variables of patients with haemophilia. These variables influence patients’ experiences of pain, catastrophism and kinesiophobia. Similarly, the clinical situation and psychosocial variables influence the quality of life perceived by these patients.

The absence of a relationship between the patients’ joint condition and their perceived quality of life should be a reason to investigate the efficacy of interventions aimed at improving quality of life. Therapies and interventions aimed at improving pain, catastrophism or kinesiophobia, such as motor imagery or movement visualization, could be useful alternatives in the management of these patients.

This study has several limitations. The first is that all subjects were recruited in Spain; consequently, these data should be extrapolated taken with caution because in subjective matters such as the perception of quality of life or pain, sociocultural components have a great influence. Therefore, it would be desirable to conduct similar studies in other countries to see if the results are comparable. Second, in this study, only patients with haemophilia A and B were recruited. Therefore, these results cannot be extrapolated to patients with other coagulopathies. Finally, this cross-sectional study only indicates the situation of the population at a given time. Future measurements with the same sample should generate a more reliable view of the patients’ situation, their perceptions over time and the evolution of the disease.

## Conclusions

The quality of life perceived by patients with haemophilia is lower than that reported by the Spanish population and by patients in other European countries. Pain, kinesiophobia and catastrophism are the main psychosocial variables that are related to a worse quality of life. Clinical variables, such as haemophilia severity and the use of drugs for pain control, are also related to a worse quality of life.

## Data Availability

The datasets used and/or analysed during the current study are available from the corresponding author on reasonable request.
